# Supplementary material for: Pyruvate Dehydrogenase Kinase 1 inhibition mediated oxidative phosphorylation enhancement in cartilage promotes osteoarthritis progression
Source: BMC Musculoskelet Disord. 2023 Jul 20;24:597. doi: 10.1186/s12891-023-06585-6 (PMC10357736; doi:10.1186/s12891-023-06585-6)
Supplement: Supplementary file 1 — Additional file 1. [file 12891_2023_6585_MOESM1_ESM.zip › MF.Gsea.1653627316242/GOMF_GLYCOLIPID_BINDING.html]

Details for gene set GOMF\_GLYCOLIPID\_BINDING[GSEA]

|  || Dataset | 55457\_collapsed\_to\_symbols.cls.cls #OA\_versus\_normal.cls.cls #OA\_versus\_normal\_repos |
| Phenotype | cls.cls#OA\_versus\_normal\_repos |
| Upregulated in class | OA |
| GeneSet | GOMF\_GLYCOLIPID\_BINDING |
| Enrichment Score (ES) | 0.38568363 |
| Normalized Enrichment Score (NES) | 1.3183299 |
| Nominal p-value | 0.106235564 |
| FDR q-value | 0.38948038 |
| FWER p-Value | 1.0 |
Table: GSEA Results Summary

  

Fig 1: Enrichment plot: GOMF\_GLYCOLIPID\_BINDING      
 Profile of the Running ES Score & Positions of GeneSet Members on the Rank Ordered List

  

| SYMBOL | TITLE | RANK IN GENE LIST | RANK METRIC SCORE | RUNNING ES | CORE ENRICHMENT || 1 | GPAA1 | glycosylphosphatidylinositol anchor attachment 1 [Source:HGNC Symbol;Acc:HGNC:4446] | 44 | 1.006 | 0.1369 | Yes |
| 2 | TREM2 | triggering receptor expressed on myeloid cells 2 [Source:HGNC Symbol;Acc:HGNC:17761] | 342 | 0.726 | 0.2152 | Yes |
| 3 | HSPA2 | heat shock protein family A (Hsp70) member 2 [Source:HGNC Symbol;Acc:HGNC:5235] | 662 | 0.603 | 0.2745 | Yes |
| 4 | PPT1 | palmitoyl-protein thioesterase 1 [Source:HGNC Symbol;Acc:HGNC:9325] | 1042 | 0.508 | 0.3160 | Yes |
| 5 | CLIP3 | CAP-Gly domain containing linker protein 3 [Source:HGNC Symbol;Acc:HGNC:24314] | 1364 | 0.448 | 0.3537 | Yes |
| 6 | MPPE1 | metallophosphoesterase 1 [Source:HGNC Symbol;Acc:HGNC:15988] | 1675 | 0.402 | 0.3857 | Yes |
| 7 | PIGK | phosphatidylinositol glycan anchor biosynthesis class K [Source:HGNC Symbol;Acc:HGNC:8965] | 2801 | 0.270 | 0.3360 | No |
| 8 | CD1C | CD1c molecule [Source:HGNC Symbol;Acc:HGNC:1636] | 3354 | 0.216 | 0.3232 | No |
| 9 | CLN6 | CLN6 transmembrane ER protein [Source:HGNC Symbol;Acc:HGNC:2077] | 3678 | 0.188 | 0.3243 | No |
| 10 | TPP1 | tripeptidyl peptidase 1 [Source:HGNC Symbol;Acc:HGNC:2073] | 3735 | 0.182 | 0.3455 | No |
| 11 | SELP | selectin P [Source:HGNC Symbol;Acc:HGNC:10721] | 4573 | 0.107 | 0.2954 | No |
| 12 | THY1 | Thy-1 cell surface antigen [Source:HGNC Symbol;Acc:HGNC:11801] | 4985 | 0.074 | 0.2738 | No |
| 13 | IL2 | interleukin 2 [Source:HGNC Symbol;Acc:HGNC:6001] | 5819 | 0.007 | 0.2102 | No |
| 14 | MANF | mesencephalic astrocyte derived neurotrophic factor [Source:HGNC Symbol;Acc:HGNC:15461] | 6170 | -0.018 | 0.1855 | No |
| 15 | LYN | "LYN proto-oncogene, Src family tyrosine kinase [Source:HGNC Symbol;Acc:HGNC:6735]" | 6514 | -0.043 | 0.1650 | No |
| 16 | GLTP | glycolipid transfer protein [Source:HGNC Symbol;Acc:HGNC:24867] | 7113 | -0.089 | 0.1310 | No |
| 17 | CEACAM5 | CEA cell adhesion molecule 5 [Source:HGNC Symbol;Acc:HGNC:1817] | 7593 | -0.127 | 0.1114 | No |
| 18 | CLN3 | "CLN3 lysosomal/endosomal transmembrane protein, battenin [Source:HGNC Symbol;Acc:HGNC:2074]" | 8094 | -0.163 | 0.0953 | No |
| 19 | SELL | selectin L [Source:HGNC Symbol;Acc:HGNC:10720] | 8319 | -0.179 | 0.1030 | No |
| 20 | CLEC4E | C-type lectin domain family 4 member E [Source:HGNC Symbol;Acc:HGNC:14555] | 9064 | -0.237 | 0.0783 | No |
| 21 | FCGR3B | Fc fragment of IgG receptor IIIb [Source:HGNC Symbol;Acc:HGNC:3620] | 9341 | -0.259 | 0.0929 | No |
| 22 | DPEP1 | dipeptidase 1 [Source:HGNC Symbol;Acc:HGNC:3002] | 9353 | -0.260 | 0.1283 | No |
| 23 | MAG | myelin associated glycoprotein [Source:HGNC Symbol;Acc:HGNC:6783] | 9490 | -0.273 | 0.1558 | No |
| 24 | PSAP | prosaposin [Source:HGNC Symbol;Acc:HGNC:9498] | 12569 | -0.780 | 0.0257 | No |
Table: GSEA details [plain text format]

  

Fig 2: GOMF\_GLYCOLIPID\_BINDING      
 Blue-Pink O' Gram in the Space of the Analyzed GeneSet

  

Fig 3: GOMF\_GLYCOLIPID\_BINDING: Random ES distribution      
 Gene set null distribution of ES for **GOMF\_GLYCOLIPID\_BINDING**
